# Soluble receptor for advanced glycation end products as an indicator of pulmonary vascular injury after cardiac surgery

**DOI:** 10.1186/1471-2466-13-76

**Published:** 2013-12-16

**Authors:** Pieter R Tuinman, Alexander D Cornet, Maria T Kuipers, Alexander P Vlaar, Marcus J Schultz, Albertus Beishuizen, AB Johan Groeneveld, Nicole P Juffermans

**Affiliations:** 1 Department of Intensive Care Medicine and Laboratory of Experimental Intensive Care and Anesthesiology (L.E.I.C.A.), Academic Medical Center, Meibergdreef 9, Amsterdam 1105, AZ, The Netherlands; 2 Department of Intensive Care Medicine, VU University Medical Center, De Boeleaan 1117, Amsterdam 1081, HZ, The Netherlands; 3 Department of Intensive Care Medicine, Erasmus MC, 's-Gravendijkwal 230, Rotterdam 3015, CE, The Netherlands; 4 Department of Intensive Care Medicine, Academic Medical Center, Room G3-227, Meibergdreef 9, 1105, AZ Amsterdam, the Netherlands

**Keywords:** sRAGE, Cardiac surgery, Transfusion, Critically ill, Acute lung injury, ARDS, Pulmonary leakage index

## Abstract

**Background:**

Cardiac surgery is frequently complicated by an acute vascular lung injury and this may be mediated, at least in part, by the (soluble) receptor for advanced glycation end products (sRAGE).

**Methods:**

In two university hospital intensive care units, circulating sRAGE was measured together with the ^68^Gallium-transferrin pulmonary leak index (PLI), a measure of pulmonary vascular permeabiliy, in 60 consecutive cardiac surgery patients stratified by the amount of blood transfusion, within 3 hours of admission to the intensive care.

**Results:**

Cardiac surgery resulted in elevated plasma sRAGE levels compared to baseline (315 ± 181 vs 110 ± 55 pg/ml, P = 0.001). In 37 patients the PLI was elevated 50% above normal. The PLI correlated with sRAGE (r^2^ = 0.11, P = 0.018). Plasma sRAGE discriminated well between those with an elevated PLI and those with a normal PLI (area under the operator curve 0.75; P = 0.035; 95% CI 0.55-0.95), with 91% sensitivity but low specificity of 36% at a cutoff value of 200 pg/mL. Blood transfusion did not influence sRAGE levels.

**Conclusions:**

sRAGE is elevated in plasma after cardiac surgery and indicates increased pulmonary vascular permeability. The level of sRAGE is not affected by transfusion.

## Background

Cardiac surgery is frequently complicated by acute lung injury (ALI) which, together with postoperative atelectasis, may prolong length of stay in the intensive care unit (ICU) [1]. ALI following cardiac surgery may be associated with trauma, use of cardiopulmonary bypass, blood transfusions, mechanical ventilation and others [1,2]. The pulmonary leak index (PLI) can be used as a measure of alveolocapillary permeability, which plays a pivotal role in the pathogenesis of ALI/acute respiratory distress syndrome (ARDS) in critically ill patients [3], for instance after cardiac surgery [4].

The mechanism for endothelial changes in increased alveolocapillary permeability after cardiac surgery remains unknown. The receptor for advanced glycation end products (RAGE) is a multi-ligand cell-surface receptor, that may be involved in alveolocapillary inflammation [5]. Some of its ligands form in the presence of hyperglycaemia or oxidant stress and are termed advanced glycation endproducts (AGE). RAGE-ligand interactions propagate inflammatory responses [6]. RAGE is expressed in systemic vascular endothelium and nervous tissues, but the highest basal levels of RAGE are displayed by alveolar epithelial type 1 cells [7]. Accordingly, soluble RAGE (sRAGE) has been postulated as a potential plasma marker of type I cell injury [8-11]. In ALI, sRAGE is released into the alveolar and plasma compartments [8] and high circulating levels are associated with severe ALI/ ARDS [9,10]. Also, cardiopulmonary bypass is associated with elevated plasma sRAGE levels [12], as is blood transfusion [11]. AGEs may form in stored red blood cells and may ligate RAGE expressed on endothelial cells, resulting in oxidative damage *in vitro*[13].

To elucidate the mechanistic role of sRAGE in alveolocapillary injury after cardiac surgery, we hypothesized that plasma sRAGE elevation following cardiac surgery is associated with increased pulmonary vascular leakage. Additionally, we investigated the role of blood transfusion as a determinant of plasma sRAGE.

## Methods

### Setting

The study is a secondary analysis of samples from a prospective case–control study (Medical Ethical Commission Academic Medical Center, Amsterdam, The Netherlands, MEC07/098#07.17.0539) in the mixed medical-surgical ICUs of two university hospitals in The Netherlands performed in 2006–2009 [14]. With approval from the ethical committee, patients 18 years or older were asked written informed consent for participation in the study prior to valvular and/or coronary artery surgery. Exclusion criteria were off-pump surgery, emergency surgery and the use of immunosuppressive drugs.

### Design

For PLI measurements, a nested case-cohort study was performed. Cardiac surgery patients were included for analysis after they had received no blood product transfusion perioperatively (n = 20), a limited transfusion regimen of 1–2 transfusions (n = 20) or multiple transfusions (> 2 units of red blood cells, 2 units of fresh frozen plasma (FFP) and 1 unit of platelets pooled from 5 donors (n = 20). Blood samples were taken before surgery and within 3 hours post operatively. In these 60 patients, within 3 hours post operatively, a PLI measurement and non-directed bronchoalveolar lavage (NBL) were performed.

### Cardiothoracic surgery/anesthesia procedures

Patients were anesthetized according to local protocol, with lorazepam, etomidate, sufentanil, and rocuronium for induction of anesthesia and sevoflurane plus propofol for maintainance of anesthesia. Steroids were given at the discretion of the cardio-anesthesiologist. As part of standard care, a pulmonary artery catheter was inserted for peri-operative monitoring. In all patients, cardiopulmonary bypass was performed under mild to moderate hypothermia (28°C-34°C), using a membrane oxygenator and a non-pulsatile blood flow. During the procedure, lungs were deflated. After the procedure, all patients were transferred to the ICU with mechanical ventilation using a pressure controlled mode with tidal volumes targeted at 6 ml/kg and positive end-expiratory pressure (PEEP). In the ICU adaptive support ventilation was used for automatic weaning in the postoperative phase. Red blood cells were transfused to maintain the haemoglobin concentration above 5.0 mmol/L (8.7 g/dL), Platelets and FFP were transfused in the case of (suspected) bleeding. Transfusions administered in the operation room or within the first 3 hours postoperatively were included in the analysis.

### Patient data collection

Potential risk factors for ALI (and therefore an increased PLI) were scored, including diabetes, smoking and alcohol abuse. Some known risk factors for ALI, such as pneumonia, trauma and sepsis were not taken into account as the patients included were elective surgery patients. For this reason, presence of such a risk factor was a reason to cancel surgery in these patients. Also, cardiopulmonary function was scored. Data on operation-time, clamp-time and hours of mechanical ventilation on the ICU were recorded in the electronic patient data system. Cardiogenic pulmonary edema was identified when pulmonary arterial occlusion pressure was > 18 mmHg (during the study period all patients received a pulmonary artery catheter pre-operatively). Chest radiographs routinely taken before surgery and on arrival at the ICU were scored for the presence of new onset bilateral interstitial abnormalities by two independent physicians who were blinded to the predictor variables. Left ventricular function was categorized as preserved (ejection fraction (EF) > 45%) or reduced (EF ≤ 45%), from pre-operative routine echocardiograms.

### Pulmonary leakage index (PLI)

For the measurement of the PLI, as described previously [15], transferrin was labelled *in vivo*, after i.v. injection of ^67^Gallium (Ga)-citrate, 4 to 5 MBq (physical half-life 78 h; Mallinckrodt Diagnostica, Petten, The Netherlands). Patients were in the supine position and two scintillation detection probes (Eurorad C.T.T., Strasburg, France) were positioned over the right and left lung apices. Starting at the time of the i.v. injection of ^67^Ga, radioactivity was detected during 30 min. The ^67^Ga counts are corrected for background radioactivity, physical half-life, spillover of ^67^Ga, obtained by *in vitro* measurement of ^67^Ga, and expressed as cpm per lung field. At 0, 5, 8, 12, 16, 20, 25 and 30 min after ^67^Ga injection, blood samples (2 ml aliquots) were taken. Each blood sample was weighed and radioactivity was determined with a single-well well-counter (LKB Wallac 1480 WIZARD, Perkin Elmer, Life Science, Zaventem, Belgium), taking background, spillover of ^67^Ga and decay into account. Results are expressed as cpm g^–1^. For each blood sample, a time-matched cpm over each lung was taken. The radioactivity ratio was calculated as (^67^Ga_lung_)/(^67^Ga_blood_) and plotted against time. The PLI was calculated from the slope of increase of the radioactivity ratio divided by the intercept, to correct for physical factors in radioactivity detection. The PLI represents the transport rate of ^67^Ga-transferrin from the intravascular to the extravascular space of the lungs and is therefore a measure of pulmonary vascular permeability. The values for both lung fields are averaged. The upper limit normal for the PLI is 14.7x10^–3^ min^–1^ and the measurement error is ~10%.

### Non-directed bronchoalveolar lavage technique

Non-directed bronchoalveolar lavage was performed by instilling 20 ml of sterile 0.9% saline via a standard 50 cm, 14 gauge tracheal suction catheter as described previously [16,17]. In short, the distal end of the catheter was introduced via the endotracheal tube and advanced until significant resistance was encountered. Immediately after instillation over 10–15 seconds, fluid was aspirated before withdrawal of the catheter. Generally, 5–10 ml of fluid was recovered.

### Assays

BALF and blood samples were centrifuged and stored at -80°C until assays were performed. In plasma and BALF, levels of sRAGE were determined by an ELISA developed in our laboratory [18]. In short, 96-well plates were coated overnight with mouse anti-human RAGE antibody (R&D systems, Minneapolis, Minnesota, USA). Samples diluted as appropriate were added and incubated for 2 hours. Next, biotinylated goat anti-human RAGE antibody (R&D Systems) was added and incubated for another 2 hours. Streptavidin poly-HRP was added for 30 minutes. Finally, sodium-acetate buffer (pH 5.5) containing 100 microg/ml tetramethylbenzidine and 0.003% H₂0₂ was added and the colour reaction was stopped by MH₂SO₄. All measurements were made in duplicate.

### Statistical analysis

Data were checked for distribution by Kolmogorov-Smirnov test. Normally distributed data were analyzed using Students t test. Non-parametric data were analyzed with Mann–Whitney U test. Categorical data were analyzed with the chi-square test. Linear correlation coefficients are reported where appropriate by Spearman correlation. To evaluate independent causal factors for an increase in PLI, a binary logistic regression analysis, using the Enter method, was applied. Statistically and/or clinically relevant factors were added to the model. The model was evaluated using Hosmer-Lemeshow goodness-of-fit test.

Data are reported as mean (± SD) or median (IQR) when appropriate. Receiver-operating-characteristic (ROC) curve was determined, the area under the curve and sensitivity and specificity derived. Statistical significance was defined as P < 0.05 and exact P values are given unless <0.001. Statistical analysis was performed with SPSS 18.0

## Results

### Patient characteristics

Patients’ baseline and peri-operative characteristics are given in Table 1. Surgery was uneventful in all patients, without the need for rethoracotomy.

**Table 1:** Demographic and peri-operative characteristics in cardiac surgery patients according to pulmonary leakage index (PLI)

	**PLI < 1.5 (n = 23)**	**PLI > 1.5 (n = 37)**	**P**
Age, years^Φ^	66 (±14)	71 (±8.5)	0.43
Sex, male^†^	19 (82)	25 (68)	0.12
EuroSCORE^Φ^	4.9 (±3.2)	7.3 (±1.5)	0.45
ASA-score^Φ^	2.7 (±0.6	2.3 (±1.2)	0.67
Diabetes mellitus^†^	4 (17)	6 (16)	0.82
COPD^†^	2 (9)	3 (8)	0.87
Left ventricular function^†^			0.65
Reduced	8 (35)	10(27)	
Preserved	15 (65)	23 (62)	
FEV1^Φ^	80 (±34)	103 (±20)	0.16
**Surgery data**			
CABG^†^	15 (65)	15 (40)	0.06
Valve replacement^†^	1 (4)	9 (24)	0.09
Other type of surgery^†^	7 (30)	13 (35)	0.27
Clamp time, min^Φ^	77 (±35)	87 (±29)	0.20
Pump time, min^Φ^	114 (±48)	114 (±33)	0.26
Operation-time, min^Φ^	292 (±81)	333 (±27)	0.02
**Perioperative data**			
Red blood cells, units^Φ^	1.5 (±3.4)	2.7 (±3.2)	0.36
Fresh frozen plasma, units^Φ^	1.2 (± 2.4)	1.2 (±2.1)	0.93
Platelets, units^Φ^	0.4 (±0.6)	0.6 (±0.8)	0.13
**Postoperative data**			
CVP, mmHg^Φ^	11 (±5)	12 (±6)	0.35
Cardiac output, liters/min^#^	5.1 (1.1)	4.3 (0.3)	0.23
Mechanical ventilation, hours^Φ^	11.7 (±6.2)	16.9 (±25.8)	0.33
PaO₂/FiO₂ ratio^#^	336 (162)	280 (112)	0.02

PLI was dichotomized into strongly elevated and modestly elevated, at a cut-off of 1.5 x elevated above upper limit of normal. EuroSCORE: European System for Cardiac Operative Risk Evaluation; ASA-score: physical status classification system according to the American Society of Anesthesiologists; FEV1: forced expiratory volume in 1 second, given in% of predicted; CVP: central venous pressure, measured at time of PLI measurement; Cardiac output and PaO2/FiO2 ratio idem; Data are presented in ^†^counts (percents), ^Φ^mean (±SD) or in ^#^median (IQR) when appropriate.

### PLI and sRAGE

**Figure 1:**
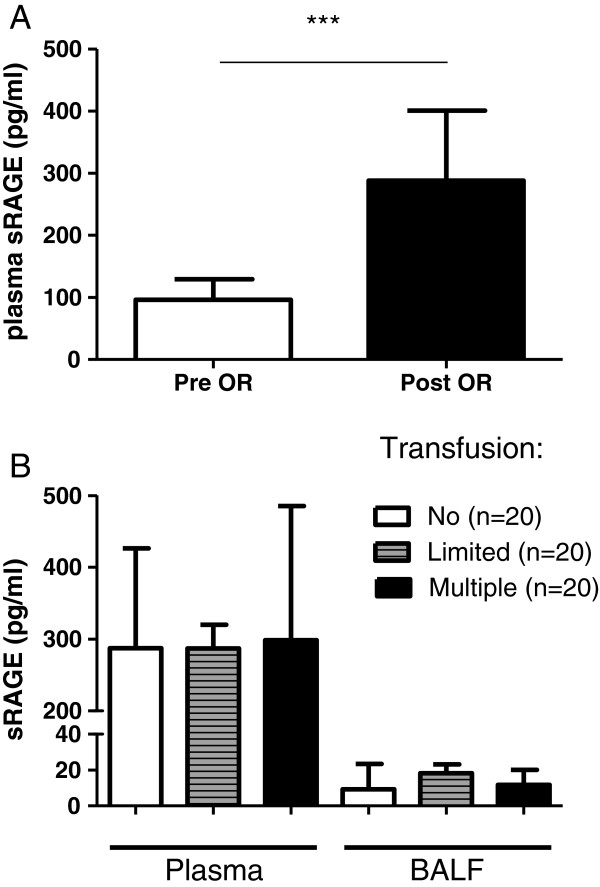
**A: Levels of sRAGE in plasma before and after cardiac surgery. B**: Levels of sRAGE in plasma BALF after surgery according to amount of transfused products. Non: non-transfused patients; Limited: 1–2 units of blood transfused; Multiple: ≥ 5 units of blood transfused. *** P < 0.001. Data are presented as median + interquartile range.

**Figure 2:**
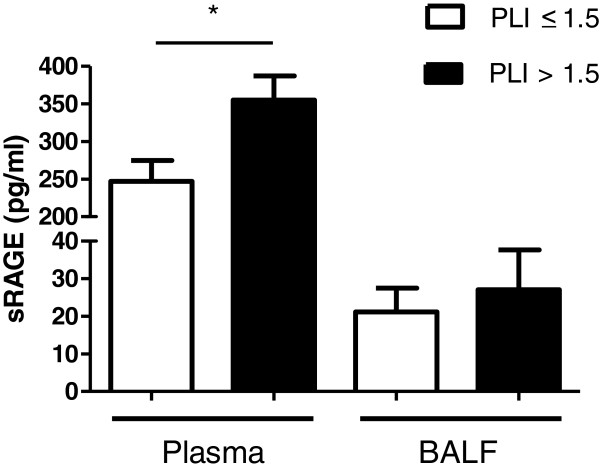
**sRAGE in plasma and BALF after cardiac surgery in patients with a pulmonary leakage index (PLI) of ≤ 1.5 or > 1.5 times the upper limit of normal (< 14.1 x 10**^**-3**^ **min**^**-**^1); *P =0.038. Data are presented as median + interquartile range.

Cardiac surgery resulted in an elevated PLI with a mean of 30 ± 18 (normal value < 14.1) x 10^-3^ min^-^1. In addition, postoperative plasma sRAGE levels were increased compared to baseline (Figure 1A). Levels of sRAGE did not differ according to stratification by the number of blood transfusions (Figure 1B). Following surgery, levels of sRAGE in BALF were more than 10-fold lower than in plasma (12 (15) vs 287 (192) pg/mL). Patients were arbitrarily divided in a group with a strongly elevated PLI (>1.5 x upper limit of normal) and a less elevated PLI (<1.5 x upper limit of normal). The group with high PLI had a lower PaO₂/FiO₂-ratio than the group with lower PLI (Table 1). Patients with a more elevated PLI had a longer duration of surgery, but not of clamp- and pump time. Patients with a strongly elevated PLI (>1.5 x upper limit of normal) had a higher level of sRAGE in plasma compared to those with a less elevated PLI (Figure 2). The levels of sRAGE in BALF did not differ between patients with a strongly elevated or less elevated PLI. There was a small but positive relation between plasma sRAGE and PLI (r 0.32, r^2^ = 0.11, P = 0.018) (Additional file 1: Figure S3). The area under the curve of the ROC-curve applied to plasma sRAGE and PLI was 0.75 (P = 0.035; 95% CI 0.55-0.95), which represents acceptable discrimination (Additional file 2: Figure S4). For plasma sRAGE, a cut-off value of 200 pg/mL was found to have a good sensitivity of 91% but low specificity of 36% for an elevated PLI.

For exploration of confounding variables, a logistic regression analysis was performed. Besides sRAGE, operation-time, EuroSCORE and amount of blood products were added to the model. Analysis showed that sRAGE and operation time were risk factors for a strongly increased PLI (Table 2). Of note, an OR of 1.005 for sRAGE applies to each increment of 1 pg/ml. Thereby, a patient with an sRAGE level of 190 pg/mL (which is the median increase in sRAGE in this population) has an OR of 2.59 (95% CI 1.47-3.71) for an increased PLI.

**Table 2:** Logistic regression analysis of risk factors in cardiac surgery patients for an increased pulmonary leakage index > 1.5 times the upper limit of normal

**Risk factors**	**OR (95%)**	***p *** **Value**
sRAGE, pg/mL	1.005 (1.00-1.01)	0.039
Operation time, min	1.010 (1.00-1.02)	0.030
EuroSCORE	1.090 (0.88-1.36)	0.441
Total amount of transfusions, units	0.968 (0.86-1.09)	0.579

PLI was dichotomized into strongly elevated and modestly elevated, at a cut-off of 1.5 x elevated above upper limit of normal. (14.1 x 10^-3^ min^-^1).

sRAGE: soluble receptor for advanced glycation end products; EuroSCORE: European System for Cardiac Operative Risk Evaluation.

Patients with bilateral pulmonary changes on chest X-ray at admission to the ICU (n = 5) had a significant lower P/F ratio compared to patients without these changes (n = 54) (188 (60) vs 315 (105), P = 0.025), as expected. In addition, in these patients levels of BALF sRAGE were elevated compared to patients with non-significant pulmonary changes on chest X-ray (97 (168) vs 18 (21) pg/mL, P <0.001). Also, PLI was more elevated in these patients (45 (30) vs 28 (16) x 10^-3^ min^-^1, P =0.050). As groups differed in operation time, which may be a confounder in the increase in sRAGE, patients were divided according to median operation time and re-analyzed. No difference was found in the median level of plasma sRAGE stratified for operation time (267 (205) vs 298 (201) pg/ml. resp., P = 0.191).

## Discussion

This study suggests that sRAGE levels, a biomarker of pulmonary tissue damage, are elevated in plasma after cardiac surgery and may serve as an indicator of pulmonary vascular injury, independent of blood product transfusion. Although sRAGE levels are increased after cardiac surgery, a mediating role of endogenous AGES in alveocapillary inflammation cannot be concluded from our results altogether.

Plasma levels of sRAGE were enhanced after lung transplantation and in ALI/ARDS, and directly associated with a poor clinical outcome [8,10]. In addition, an increase in plasma sRAGE was found in cardiac surgery patients after cardiopulmonary bypass [12]. We now demonstrate that sRAGE is linked to increased pulmonary capillary permeability and a decrease in oxygenation, suggesting a possible mediator role in lung injury after cardiac surgery. The higher plasma sRAGE levels were associated with a more elevated PLI, which is an established early marker of increased capillary permeability in the lung [15], so that the elevated plasma sRAGE and PLI may both reflect endothelial injury in the lungs occurring during cardiac surgery, even before the clinical criteria of ALI are met [19]. The possible diagnostic value of sRAGE in lung injury found in our study may be specific for cardiac surgery patients, since sRAGE was recently found not to be predictive of lung injury in other critically ill patients [20]. However, our results are in line with a previous study in children which found plasma sRAGE to enable prediction of acute lung injury after cardiac surgery [21]. We hypothesize that during progression of lung injury, sRAGE production by alveolar cells may become more prominent and higher levels of sRAGE in BALF may be found as suggested before [8], since we found higher levels of sRAGE in BALF in patients with marked pulmonary findings on chest X-ray. In line with reflecting lung vascular injury, patients with a more elevated PLI had a lower PaO₂/FiO₂ ratio and longer duration of mechanical ventilation.

In contrast to the association between the volume of blood transfused and the plasma levels of sRAGE [11], we did not observe a relation between blood product transfusion and sRAGE, suggesting that upregulation and shedding of the receptor in pulmonary endothelium was caused by endogenously released AGE. Conversely, the use of cardiopulmonary bypass elevating sRAGE may have been instrumental, as suggested before [11,12,22]. Alternatively, the rise in plasma sRAGE may be a reflection of alveolar epithelial cell injury, possibly caused in part by deflation of the lungs during surgery. Since in our patients plasma levels were 10-fold higher then in BALF, a systemic production of sRAGE seems more likely then alveolar sRAGE production in this study, unless mainly involving pulmonary endothelium and intravascular shedding. Of note, one must take into account that the BALF samples are diluted to some account.

This study may have implications for future efforts to decrease lung injury following cardiac surgery. Previous experimental data suggest that sRAGE may neutralize the ligand-mediated damage by acting as a decoy, thus protecting sensitive cells from the deleterious effects of ligand-RAGE hyperactivity [23,24]. In line, blocking of RAGE-ligand interaction by recombinant sRAGE was protective in murine models of lung injury [25] and ischemic heart injury [26]. Furthermore, determining sRAGE early in the course of lung injury may be of therapeutic significance, because patients with higher sRAGE levels have been shown to benefit the most from lung protective mechanical ventilation [9]. Our results contribute to the rationale of exploring the therapeutic potential of RAGE as an interventional target in patients at risk of developing ALI/ARDS.

This study has several potential limitations. Although we corrected for possible confounding by performing a logistic regression analysis and by dividing patients according to operation time, we can not exclude confounders unaccounted for. In addition, we performed an in house ELISA and levels were about 4 times lower than those measured in studies using commercially available kits [12]. However, levels were comparable to a previous study in our centre [18]. Furthermore, a comparison of sRAGE to other biomarkers of lung injury (for example surfactant protein B, which leaks in the bloodstream because of alveolar-capillary damage) [27], would have strengthened our data.

## Conclusions

sRAGE is elevated in plasma after cardiac surgery and indicates increased pulmonary vascular permeability. The level of sRAGE is not affected by transfusion.

## Abbreviations

AGE: Advanced glycation endproducts; ALI: Acute lung injury; ARDS: Acute respiratory distress syndrome; CI: Confidence interval; FFP: Fresh frozen plasma; ICUs: Intensive care units; IQR: Interquartile range; NBL: Non-directed bronchoalveolar lavage; OR: Odds ratio; PLI: Pulmonary leakage index; SD: Standard deviation; sRAGE: Soluble receptor for advanced glycation end products.

## Competing interests

The authors declare that they have no competing interests.

## Authors’ contributions

PRT: was instrumental in developing the study hypothesis and was intimately involved in interpretation of the results as well as manuscript preparing. He was also involved in data extraction as well as statistics. He has full access to the data, read the final version of the manuscript and agrees with all reported findings and interpretations. ADC: was instrumental in the performance of coordination of the study, data gathering and analysis. He has revised the manuscript critically for important intellectual content, read the final version of the manuscript and agrees with all reported findings and interpretations. MTK: was instrumental in the performance of laboratory analysis. She was also intimately involved with interpretations of the results and manuscript. She has revised the manuscript critically for important intellectual content, read the final version of the manuscript and agrees with all reported findings and interpretations. APV: was instrumental in the performance of data gathering. He was also intimately involved with interpretations of the results and manuscript. He has read the final version of the manuscript and agrees with all reported findings and interpretations. Furthermore, he is the archival author. MJS: was instrumental in study hypothesis and design. He has revised the manuscript critically for important intellectual content, read the final version of the manuscript and agrees with all reported findings and interpretations. AB: was instrumental in study hypothesis and design. He has revised the manuscript critically for important intellectual content, read the final version of the manuscript and agrees with all reported findings and interpretations. JBG: was instrumental in study hypothesis and design. He was intimately involved in manuscript preparing. He has read the final version of the manuscript and agrees with all reported findings and interpretations. NPJ: was instrumental in developing study hypothesis and was intimately involved in interpretation of the results as well as manuscript preparing and data statistics. She has read the final version of the manuscript and agrees with all reported findings and interpretations. All authors read and approved the final manuscript.

## Pre-publication history

The pre-publication history for this paper can be accessed here:

http://www.biomedcentral.com/1471-2466/13/76/prepub

## Supplementary Material

Additional file 1: Figure S3 The receiver operating curve of the ability of sRAGE plasma levels after cardiac surgery to predict the development of a strongly increased pulmonary leakage index of > 1.5 times the upper limit of normal (< 14.1 x 10^-3^ min^-^1). Click here for file

Additional file 2: Figure S4 Scatterplot of the relation between pulmonary leakage index (PLI) and plasma levels of soluble receptor of advanced glycation end products (sRAGE). Click here for file
