# Supplementary material for: Soluble receptor for advanced glycation end products as an indicator of pulmonary vascular injury after cardiac surgery
Source: BMC Pulm Med. 2013 Dec 16;13:76. doi: 10.1186/1471-2466-13-76 (PMC3866278; doi:10.1186/1471-2466-13-76)
Supplement: Additional file 1: Figure S3 — The receiver operating curve of the ability of sRAGE plasma levels after cardiac surgery to predict the development of a strongly increased pulmonary leakage index of > 1.5 times the upper limit of normal (< 14.1 x 10-3 min-1). [file 1471-2466-13-76-S1.doc]

**Figure 3.** The receiver operating curve of the ability of sRAGE plasma levels after cardiac surgery to predict the development of a strongly increased pulmonary leakage index of > 1.5 times the upper limit of normal (< 14.1 x 10^-3^min^-^1).
