# Supplementary material for: Soluble receptor for advanced glycation end products as an indicator of pulmonary vascular injury after cardiac surgery
Source: BMC Pulm Med. 2013 Dec 16;13:76. doi: 10.1186/1471-2466-13-76 (PMC3866278; doi:10.1186/1471-2466-13-76)
Supplement: Additional file 2: Figure S4 — Scatterplot of the relation between pulmonary leakage index (PLI) and plasma levels of soluble receptor of advanced glycation end products (sRAGE). [file 1471-2466-13-76-S2.doc]

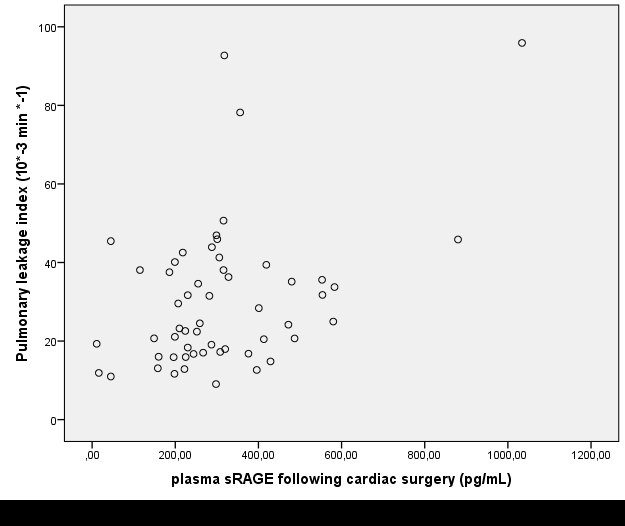


Figure 4. Scatterplot of the relation between pulmonary leakage index (PLI) and plasma levels of soluble receptor of advanced glycation end products (sRAGE).
